# Effect of Using Eye Masks and Earplugs on the Risk of Post-traumatic Stress Disorder Development in Patients Admitted to Cardiac Surgery Intensive Care Units

**DOI:** 10.5005/jp-journals-10071-23109

**Published:** 2019-01

**Authors:** Jalil Azimian, Omid Assar, Amir Javadi, Zohreh Froughi

**Affiliations:** 1 Department of Critical Care Nursing, School of Nursing and Midwifery, Qazvin University of Medical Science, Qazvin, Iran; 2 Open-heart cardiac surgery intensive care unit, Shahid Rajaee Hospital, Alborz University of Medical Science, Karaj, Iran; 3 Social Medicine Department, Medicine Faculty, Qazvin University of Medical Science, Qazvin, Iran; 4 Student Research Committee, Qazvin University of Medical Science, Qazvin, Iran

**Keywords:** Ccritically ill patient, Incidence risk, Intensive care unit, Post-traumatic stress disorder, Psychological disorders, Critical care team members can use eye masks and earplugs as a cheap and nonpharmacological method for reduces the risk of post-traumatic stress disorder development among critically ill patient.

## Abstract

**Context:**

Critically ill patients are at risk of post-traumatic stress disorder development.

**Aim:**

The purpose of this study was to investigate the effect of using eye masks and earplugs on the risk of post-traumatic stress disorder development in patients admitted to cardiac surgery intensive care units.

**Settings and design:**

This is a clinical trial that conducted in intensive care units in Iran.

**Materials and methods:**

Sample of the present clinical trial consisted of 68 patients undergoing open-heart cardiac surgery that were randomly assigned to two groups. Patients in the control group received the usual care, and patients in the intervention group used eye masks and earplugs during sleep. The risk of post-traumatic stress disorder development before surgery and 2 months after discharge from the intensive care unit was assessed using the impact of event scale-revised.

**Statistical analysis:**

Statistical analysis: Data were analyzed using paired t-test and independent t-test in Statistical Package for Social Sciences (SPSS) version 24 software.

**Results:**

Patients in the two groups were similar in terms of demographic characteristics. The mean of the total scores of patients in the control and intervention group before surgery was 10.41 ± 5.25 and 10.71 ± 5.10, respectively (p = 0.82). The mean of the total scores of patients in the control and intervention group 2 months after discharge was 29.50 ± 5.90 and 11.72 ± 6.48, respectively (p <0.001).

**Conclusion:**

The use of eye masks and earplugs significantly reduces the risk of post-traumatic stress disorder (PTSD) development in patients undergoing open-heart cardiac surgery.

**How to cite this article:**

Azimian J, Assar O, Javadi A, Froughi Z. Effect of Using Eye Masks and Earplugs on the Risk of Posttraumatic Stress Disorder Development in Patients Admitted to Cardiac Surgery Intensive Care Units. Indian Journal of Critical Care Medicine, January 2019;23(1):31-34.

## INTRODUCTION

Post-traumatic stress disorder (PTSD) is originally known as "shell shock" and "gross stress reaction".^1^ PTSD is a disorder caused by trauma and is characterized by reviewing the traumatic event, avoidance of traumatic stimuli, emotional blunting, excessive tingling, and difficulty in sleeping, difficulty with memory, and lack of concentration.^2^ The person with PTSD re-experiences the traumatic event *via* intrusive memories or nightmares and these events come to mind unconsciously, and the relevant symptoms reappear when the person is in the same situation as the traumatic event.^2^ Patients admitted to intensive care units are also at the risk of experiencing psychological disorders, including PTSD, due to traumatic events.^3^ Previous studies indicate a high prevalence of this disorder among this group of patients. In a study in 2007, Girard et al. investigated the prevalence, and the risk factors of PTSD development in patients admitted to intensive care units who were in need of mechanical ventilation. The results of this study showed that about 14% of patients suffer from severe symptoms of PTSD 6 months after discharge.^4^ In another study on this matter in Iran, Sadat et al. investigated the prevalence of PTSD among patients discharged from critical care unit. The prevalence rate was reported to be 48%.^5^

Factors such as aging, female gender, increased the length of hospital stay, mechanical ventilation, drug misuse, taking certain sedative drugs, painful procedures, environmental unawareness, loss of control, environmental disruption, an imminent threat of death, delirium and sleep disturbance have all been implicated in chance of developing PTSD in a patient admitted to intensive care units.^5-8^ Sleep disturbance is one of the modifiable variables implicated in the development of PTSD which can be regulated by non-pharmacological measures so as to reduce the incidence risk of PTSD.^3^ Two of the common methods used in this regard are the use of earplugs and eye masks. Mashayekhi et al. have conducted two studies regarding this. In one study, the effects of eye masks and in the other study, the effect of earplugs on improving the sleep quality of patients in the coronary care unit (CCU) was investigated. The results of Mashayekhi et al. studies demonstrated that both eye masks and earplugs significantly improve the quality and quantity of sleep in patients in cardiac care units.^9,10^

Previous studies have shown that there is a correlation between sleep quality and the development of PTSD in patients with special health care needs.^11,12^ Due to the effectiveness of earplugs and eye masks as two non-pharmacological methods that increase the quality of sleep in patients, this hypothesis was made for researchers to determine whether the use of them can be helpful, due to sleep quality improvement, to reduce the incidence risk of PTSD in patients admitted to ICUs. Therefore, the present study was conducted with the aim of the effect of using eye masks and earplugs on the risk of PTSD development in patients admitted to cardiac surgery intensive care units.

## MATERIALS AND METHODS

The present clinical trial study was conducted in Karaj, in 2017 and 2018. The study setting was the open-heart cardiac surgery intensive care unit of Shahid Rajaee Hospital affiliated to Alborz University of Medical Sciences. This unit has 14 beds that are separated using Paravans.

### Sample and Sampling

Patients undergoing open-heart cardiac surgery, who were eligible to participate in the study, were enrolled in the study. The sample size was calculated to be 27 people in each group by using the sample size formula and considering the mean and the standard deviation scores of PTSD in the two groups of control and intervention, which were estimated to be 23 ± 7 and 6 ± 18, respectively, the confidence interval of 95% and the test power of 80%. However, the final sample size was calculated to be 34 in each group due to the drop-out rate.


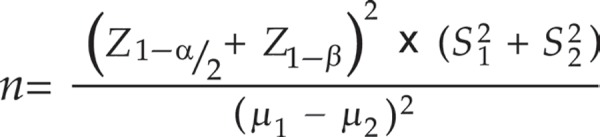


Therefore, 64 patients were selected in the study. Sampling was done randomly using balls and bag method. This was carried out by one of the nurses who were not aware of the study. The inclusion criteria were aged over 18 years old, having no history of mental illnesses, having no history of any recent stress rather than cardiac surgery and having no history of drug and alcohol addiction. Patients, who were in need of more than one surgery and those who were not willing to participate in the study were excluded. Patients undergoing emergency cardiac surgery were also excluded.

### Intervention and Data Collection

The control group in this study did not receive any intervention care rather than the usual one of the intensive care unit. In the intervention group, in addition to the usual care, patients were instructed to use earplugs and eye masks during sleep between 10 AM to 6 AM (according to the pattern used in the study area). The use of earplugs and eye masks was continued every night till the patient's discharge. Before the use of earplugs and eye masks, patients were instructed about the use of them by one of the nurses or researchers. Demographic characteristics of patients were collected by a researcher-made checklist before the study. This checklist consisted of items such as age, gender, education, job, etc. Also, the symptoms of PTSD in patients, who were willing to participate in the study, were evaluated by the researcher one day before the surgery.

Additionally, the incidence risk of PTSD was evaluated 2 months after the patient was discharged from the section. This was done by calling the patient via telephone. For this purpose, the researcher contacted him by using a contact number that had already been received and asked them the items of the questionnaire. The Impact of Event Scale-Revised (IES-R) was used to assess the PTSD. This questionnaire has 22 items. The items are divided into three subscales including intrusive memories (6 items), hyperarousal (6 items) and avoidance (8 items). Answers are based on a five-item Likert scale; not at all (score: 0), a little bit (score: 1), moderately (score: 2), quite a bit (score: 3) and extremely (score 5). Total score ranges from 0 to 88.

### Ethical Consideration

Proposal of the present study evaluated and approved by the ethics committee of Qazvin University of Medical Sciences. The study also registered in Iranian Registry of Clinical Trial (IRCTID: IRCT20180803040683N1). Before surgery, patients received information about study aims and requested to fulfill and signed a consent form. Data remained anonymous and kept confidential.

### Statistical Tests

After data collection, the findings were presented in form of statistical tables, graphs, and numerical indices. Kol mogorov-Smirnov test was used to test the normalization of quantitative data. A paired t-test was used to compare the mean of the total score of each group before and after the intervention, and independent t-test was used to compare the mean of the total score of the two groups. Data were analyzed using SPSS version 24 software. A p-value less than 0.05 considered significant.

## RESULTS

Of the 34 patients in the control group, six patients were excluded from the study due to several reasons such as death (1 case) and not returning calls (5 cases). In the intervention group, five patients were excluded due to not returning calls, as well. Patients in the two groups were similar in terms of demographic characteristics. Table 1 shows the demographic characteristics of patients in the control and intervention group in more details.

The mean of the total scores of patients in the control and intervention group before surgery was 10.41 ± 5.25 and 10.71 ± 5.10, respectively. This difference was not statically significant between the two groups (p = 0.82). Table 2 shows the mean of the total scores of patients in more details. The mean of the total scores of patients in the control and intervention group 2 months after discharge was 29.50 ± 5.90 and 11.72 ± 6.48, respectively. Based on the results of the independent t-test, this difference was statically significant between the two groups (p <0.001). Table 3 shows the mean of the total scores of patients 2 months after discharge in more details.

## DISCUSSION

Today, due to the development and advancement of technology, and care and treatment, the chance of survival for patients admitted to intensive care units have risen, and this has increased the need for more attention after discharge. One of the problems that a large percentage of these patients may be exposed after surgery is PTSDs. In this study, the effect of a non-pharmacological intervention, which was the use of earplugs and eye masks during the admission of patients in the intensive care unit, was investigated. Based on the results of this study, the use of earplugs and eye masks in preventing the development of PTSD is effective.

**Table 1:** Patients demographics characteristics

*Item*		*Intervention*	*control*	*p-value*
sex	Male	19	17	0.40
	Female	15	17	
Marital status	Single	2	6	0.099
	Married	32	26	
Surgery history	Yes	17	16	0.53
	No	17	18	
Age		58.7 ± 7. 5	57.3 ± 7. 6	0.47

**Table 2:** Comparison of the mean of the total and subscales scores of PTSD in the control and intervention group before intervention

*Before Intervention*	*Group*	*No.*	*mean*	*SD*	*p-value*
Avoidance	control	34	3.76	2.19	0.48
	intervention	34	4.18	2.58	
Intrusive memories	control	34	3.24	2.22	0.53
	intervention	34	3.56	2.02	
Hyperarousal	control	34	3.44	2.25	0.303
	intervention	34	2.94	1.69	
Total	control	34	10.41	5.25	0.82
	intervention	34	10.71	5.10	

Critically ill patients in whom PTSD develops may have serious complications. This disorder is associated with physical and psychological disorders and decreased quality of life.^2^ Precautionary measures of this disorder are so important. In this study, we investigated the use of eye masks and earplugs. Considering that the high levels of light and sound in the intensive care unit are the two important factors in sleep disturbance of ICUs patients, it seems that the use of eye masks and earplugs in patients during admission to ICUs can regulate them to some extent and improves the quality of their sleep and subsequently reduces the risk of PTSD after ICU discharge.^9,10^ Not only did not scientific literature show a similar study to the present study, but also studies on other non-pharmacological interventions to prevent the development of PTSD in ICUs patients are very limited. In a study in Italy in 2011, Peris et al. investigated the effect of early intra-ICU clinical psychologist intervention on the development of PTSD after discharge. The interventions have been done in this study were educational interventions, stress management, counseling, psychological support and coping strategies, and family counseling and education. The development of psychological disorders including PTSD was investigated after 12 months from ICU discharge. Finally, Peris et al. suggested that the use of early intra-ICU clinical psychologist intervention might reduce the incidence of psychological disorders in these groups of patients.^13^ In another study regarding this in 2014, Bethell examines the effect of a psychological protocol on the development of PTSD in patients undergoing open-heart cardiac surgery. In this study, 33 patients were recruited, and the patients in the intervention group received psychological educations in terms of prevention and identification of PTSD before and two weeks after the surgery. Similar to the present study, Bethell et al. used IES-R to investigate the development of PTSD. The results of Bethell study showed that the use of psychological educations in patients undergoing open-heart cardiac surgery could significantly reduce the risk of developing PTSD.^14^ In another study on this issue in 2010, the researchers investigated the effect of using ICU diaries on the developing of PTSD after ICU discharge. ICU diaries is a kind of diary that is written by nurses and patients' families in order to fill the memory gaps of those who are receiving mechanical ventilation.^15,16^ In this study, 352 patients were randomly assigned into two groups. The intervention group was the one for which ICU diaries was performed. In this study, the PTSS-14 screening tool was used to investigate PTSD. The development of PTSD was examined 3 months after discharge. The results of this study showed that ICU diaries had a significant effect on the prevention of PTSD so that the development of PTSD in the control group was >2 times higher than the intervention group (13% *vs.* 5%).^17^

**Table 3:** Comparison of the mean of the total and subscales scores of PTSD in the control and intervention group after intervention

*After Intervention*	*Group*	*No.*	*mean*	*SD*	*p-value*
Avoidance	Control	28	11.07	2.97	<0.001
	Intervention	29	4.97	3.94	
Intrusive memories	Control	28	9.39	1.85	<0.001
	Intervention	29	3.41	2.653	
Hyper arousal	Control	28	9.61	2.99	<0.001
	Intervention	29	3.34	2.26	
Total	Control	28	29.50	5.90	<0.001
	Intervention	29	11.72	6.48	

## CONCLUSION

Although the prevalence of developing PTSD after discharge is high and has various negative effects on the patient, prevention efforts have been underestimated. This is the first study conducted to test this hypothesis that an increase in sleep quality could reduce the incidence risk of PTSD after discharge by using a non-pharmaceutical method. The use of eye masks and earplugs significantly reduces the incidence risk of PTSD in patients undergoing open-heart cardiac surgery. Given the low-cost and availability of this method, it is recommended that this method be used as a common interventional method in ICUs patients. Due to lack of studies, further studies are needed in this regard. Furthermore, using these methods, which are eye masks, earplugs and ICU Diaries simultaneously can have significant outcomes. It is also recommended that the effect of using eye masks and earplugs on the incidence risk of developing PTSD be evaluated over a longer period of time.
